# Submuscular Lipoma of the Forehead

**Published:** 2015-03-24

**Authors:** Sara K Neches, Alexis L. Parcells, Adam M. Feintisch, Mark S. Granick

**Affiliations:** Division of Plastic Surgery, Rutgers New Jersey Medical School, Newark

**Keywords:** forehead lipoma, submuscular lipoma, forehead mass, subfrontalis mass, submuscular forehead mass

## DESCRIPTION

A 35-year-old man presented with a 2-cm centrally located mass on his forehead. The mass appeared soft and mobile. It had been present for several years and was slow growing. The patient denied pain or numbness.

## QUESTIONS

1. **What is the differential diagnosis for forehead masses?**
2. **What important clinical features define forehead lipomas?**
3. **How are these lesions best managed?**
4. **What is the risk of malignant transformation?**

## DISCUSSION

Common forehead masses include dermoid cysts, hemangiomas, lipomas, epidermal inclusion cysts, and osteomas.^1^ Lipomas are the most common tumor of mesenchymal origin, and frontalis-associated lipomas are based on 4 subtypes: *intramuscular*, arising from within the frontalis muscle of the forehead; *submuscular*, between the frontalis and its deep investing fascia (galea); *subgaleal*, between the galea and the periosteum; and *subperiosteal*.^2^ These masses are most commonly found in men 40 to 70 years old. They often develop independent of trauma and have no genetic basis.^2^^,^^3^

**Figure 1:**
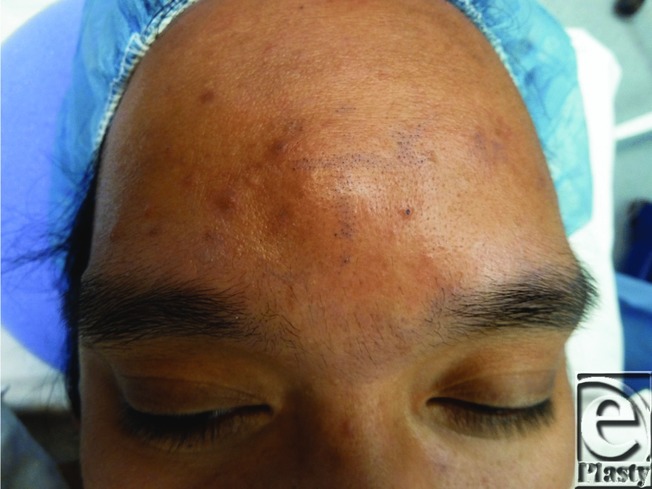
Submuscular forehead lipoma.

Forehead lipomas are diagnosed clinically. Lipomas are slow-growing, singular masses rarely exceeding several centimeters in size. Patients are often asymptomatic and deny pain or tenderness over the lesion.^2^ Subcutaneous lipomas are soft and pliable, whereas subgaleal lipomas tend to be fixed and firm^2^^,^^4^ (Fig 1). These masses are easily distinguished from the taut, fluid-filled, epidermal inclusion cyst or hardened osteomas.^2^ Diagnostic modalities including ultrasonography, computed tomography, or magnetic resonance imaging can further identify the lesion and its boundaries and aid in surgical planning.^1^

While these lesions may be managed by observation, the forehead is a cosmetically sensitive area and most patients elect for surgical excision. A minimally invasive endoscopic approach has been described for subcutaneous lipomas to reduce scarring, avoid injury to the supraorbital and supratrochlear neurovascular bundles, and reduce postoperative pain.^5^ For deeper frontalis-associated lipomas, direct en bloc resection is often required to successfully excise the tumor.^2^^,^^4^

Forehead lipomas are generally benign with no malignant potential, and excision is considered curative. However, liposarcoma must be differentiated from other benign mesenchymal tumors on the basis of histologic findings such as poorly defined margins or immature and polymorphic cells.^6^

**Figure 2:**
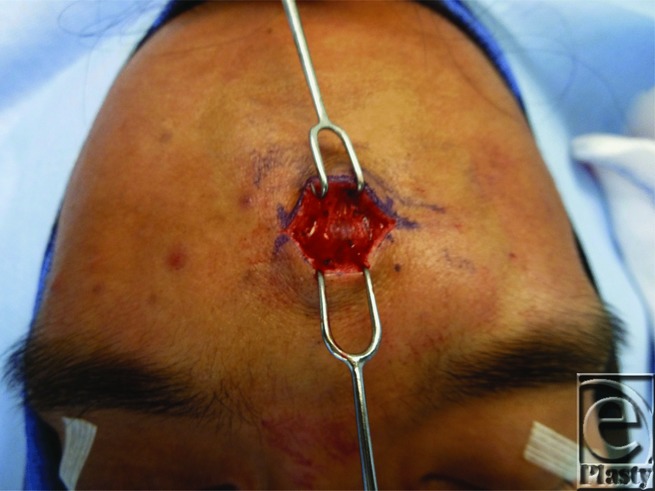
Lipoma exposed under the frontalis muscle.

**Figure 3:**
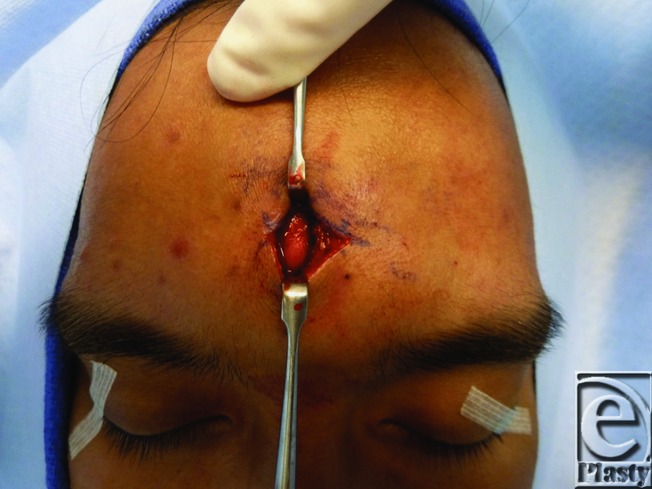
Exposed lipoma after blunt dissection.

**Figure 4:**
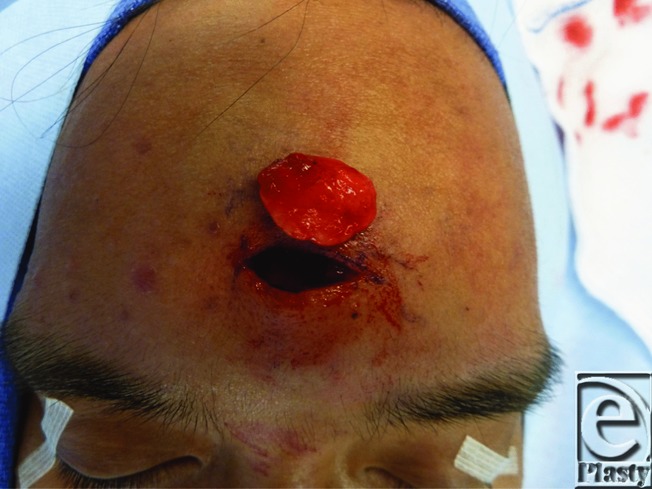
En bloc resection of the forehead lipoma.

Our patient underwent direct surgical excision. The skin was incised transversely through forehead rhytid and the dissection directed to the frontalis muscle (Fig 2), which was incised vertically to avoid neurovascular injury and identify the tumor fixed to the galea (Fig 3). Blunt dissection continued along the perimeter of the tumor to ensure complete resection (Fig 4). Pathology identified the mass as mature adipose tissue consistent with a subgaleal lipoma. To restore functional integrity of the muscle, a layered closure was performed. Any indentations or contour irregularities caused by the tumor naturally remodeled over time.
